# Study protocol for the recruitment of female sex workers and their non-commercial partners into couple-based HIV research

**DOI:** 10.1186/1471-2458-12-136

**Published:** 2012-02-20

**Authors:** Jennifer L Syvertsen, Angela M Robertson, Daniela Abramovitz, M Gudelia Rangel, Gustavo Martinez, Thomas L Patterson, Monica D Ulibarri, Alicia Vera, Nabila El-Bassel, Steffanie A Strathdee

**Affiliations:** 1 Department of Anthropology, University of South Florida, 4202 East Fowler Avenue, SOC 107, Tampa, FL 33620-7200, USA; 2 Division of Global Public Health, School of Medicine, University of California at San Diego, 9500 Gilman Drive, La Jolla, CA 92093-0507, USA; 3 El Colegio de La Frontera Norte, Carretera Escénica Tijuana -Ensenada, Km 18.5, San Antonio del Mar, 22560 Tijuana, Baja California, Mexico; 4 Salud y Desarrollo Comunitario de Ciudad Juárez A.C. (SADEC) and Federación Mexicana de Asociaciones Privadas (FEMAP), Ave. Malecón No. 788 Col. Centro C.P., 32000 Ciudad Juárez, Chihuahua, Mexico; 5 Department of Psychiatry, University of California at San Diego, 9500 Gilman Drive, La Jolla, CA 92093-0603, USA; 6 Columbia University School of Social Work, 1255 Amsterdam Avenue, New York, NY 10027, USA

**Keywords:** Methods, Recruitment, Eligibility screening, Couple-based research, Female sex workers, Mexico

## Abstract

**Background:**

Researchers are increasingly recognizing the importance of addressing sexual and drug-related HIV risk within the context of intimate relationships rather than solely focusing on individual behaviors. Practical and effective methods are needed to recruit, screen, and enroll the high risk and hard-to-reach couples who would most benefit from HIV interventions, such as drug-using female sex workers (FSWs) and their intimate, non-commercial partners. This paper outlines a bi-national, multidisciplinary effort to develop and implement a study protocol for research on the social context and epidemiology of HIV, sexually transmitted infections (STI), and high risk behaviors among FSWs and their non-commercial male partners in Tijuana and Ciudad Juarez, Mexico. We provide an overview of our study and specifically focus on the sampling, recruitment, screening, and successful enrollment of high risk couples into a public health study in this context.

**Methods/Design:**

We used targeted and snowball sampling to recruit couples through the female partner first and administered a primary screener to check her initial eligibility. Willing and eligible females then invited their primary male partners for couple-based screening using a couple verification screening (CVS) instrument adapted from previous studies. The CVS rechecked eligibility and separately asked each partner the same questions about their relationship to "test" if the couple was legitimate. We adapted the original protocol to consider issues of gender and power within the local cultural and socioeconomic context and expanded the question pool to create multiple versions of the CVS that were randomly administered to potential couples to determine eligibility and facilitate study enrollment.

**Discussion:**

The protocol successfully enrolled 214 high risk couples into a multi-site public health study. This work suggests the importance of collaborating to construct a study protocol, understanding the local population and context, and drawing on multiple sources of input to determine eligibility and verify the legitimacy of relationships. We provide a practical set of tools that other researchers should find helpful in the study of high risk couples in international settings, with particular relevance to studies of FSWs and their intimate partners.

## Background

Researchers are increasingly recognizing the importance of addressing HIV risk within the context of intimate relationships rather than solely focusing on individual behaviors [1,2]. A growing body of evidence suggests that couple-based interventions may be more efficacious than individual-based interventions in promoting safer sex behaviors [3,4] and reducing drug use [5,6]. However, a recent review of couple-based interventions cautioned that additional research is needed to build a stronger theoretical and methodological basis for couples-focused HIV interventions [7]. Practical and effective methods are needed to recruit, screen, and enroll high risk couples into studies in diverse social and cultural contexts. In particular, protocols are needed to recruit the socially marginalized and hard-to-reach couples who would most benefit from HIV interventions, such as drug-using female sex workers (FSWs) and their intimate, non-commercial partners.

This paper outlines a bi-national, multidisciplinary effort to develop and implement a study protocol for research on the context and epidemiology of HIV, sexually transmitted infections (STIs), and high risk behaviors among FSWs and their primary, non-commercial male partners in Tijuana and Ciudad Juarez, Mexico. This work includes an adaptation of a protocol developed by McMahon and colleagues (2003) [8] that recruits couples first through the female partner, and then screens both partners to verify couple status prior to enrollment. Through a detailed explanation of our methodological approach, we demonstrate the need for collaborative processes in constructing a protocol, maintaining sensitivity to the local population and socioeconomic context, and drawing on multiple sources of input to determine eligibility and verify the legitimacy of relationships in order to ensure the successful recruitment of high risk couples. We provide a practical set of tools that other researchers should find helpful in the study of high risk couples in international and resource-poor settings, with particular relevance to studies of FSWs and their intimate partners.

### Couple-based studies: sampling, recruitment, and screening

Health studies focusing on sexual relationships present a unique set of challenges for researchers, particularly when working with street-based, low income, and otherwise socially marginalized populations [4,8-12]. Diligent preparation is needed in the design and implementation of recruitment procedures for dyadic research, which is highly dependent on the local context and nature of the study [12,13], as well as the broader cultural context. Researchers must first consider their approach to sampling and recruitment, particularly when potential participants may be considered a "hidden population" because of their exclusion from mainstream health and social services.

Recruitment strategies in heterosexual couple-based research have included a variety of clinic and community-based settings [11,13], as well as targeted street outreach [8]. While some studies have recruited through both the male and female partners [14], others have recruited initially though the female partner [8,12]. Witte and colleagues (2004) [12] described a process of recruiting predominantly African-American and Latina women through an outpatient clinic using a brief screener to determine initial eligibility. Eligible women were then offered different strategies to recruit their partner. A "brokering" strategy entailed women describing the project to her partner and encouraging his participation on her own, while a "co-recruitment" strategy meant that staff assisted the female in the recruiting process by sending a letter, calling, or inviting the male partner to the project office in person to describe the study [15]. Male staff members also offered to role play with the women to help them present the study in a positive light to their partner [12].

Research with couples in which one or both partners are active drug users can add an additional layer of complexity to protocol design. El-Bassel and colleagues (2011) recently published one of the few randomized controlled trials to address both sexual and drug-related risk behaviors among low-income couples, who were recruited through street outreach, homeless shelters, soup kitchens, syringe exchange programs, and word of mouth [16]. Working with a mostly unstably housed cohort of street-based drug users, their study demonstrated reductions in risk behavior across groups and suggested the efficacy of the couple-based intervention design. Like the majority of couple-based studies, they relied on self reported drug use and relationship status, where enrollment was based on the index participant's eligibility and willingness to recruit their partner into the study, who was also individually screened. Eligible couples received monetary compensation at baseline and at each follow-up visit. While monetary reimbursements are a standard and ethical practice in drug-related studies [17], relying on unverified self-reported data for study qualification leaves researchers vulnerable to the possibility of enrolling ineligible individuals who may pose as qualified participants to obtain the compensation.

McMahon et al. (2003) [8] appear to be the first researchers to explicitly document a research protocol for recruiting and verifying the relationship status of street-based, drug-using couples into a HIV/STI prevention study. Like other couple-based studies, they used an adaptive sampling strategy to recruit through the female partner first to establish initial eligibility. As a next step, they introduced the use of a couple verification screening (CVS) instrument to prevent dyads who were not true couples from enrolling in the study. The CVS was designed to "test" the knowledge of each partner by asking the same personal questions of each partner separately and then comparing their answers as a strategy to verify relationship status. Rather than solely relying on individual self report of relationship status, screening tools like the CVS may prove invaluable in systematically excluding illegitimate couples from research studies. The authors suggested that their integrated approach to sampling, recruiting, and screening hard-to-reach couples can be adapted to other hidden populations, such as sex workers. To our knowledge, ours is the first couple-based study designed exclusively to reach FSWs and their intimate, non-commercial male partners for participation in an HIV/STI prevention project.

### Rationale for studying female sex workers and their intimate partners

Internationally, FSWs are at risk for multiple health harms, including HIV/STI transmission [18-21]. While the national HIV prevalence in Mexico remains low, prevalence is much higher among risk groups along Mexico's Northern border with the United States. HIV prevalence among FSWs in Tijuana and Ciudad Juarez has risen over the last decade from less than 1% to 6% overall and 12% among FSWs who inject drugs (FSW-IDUs). A recent study of FSWs in the region also documented a high prevalence of STIs, which can facilitate HIV transmission, including gonorrhea (6.4%), chlamydia (13%), and syphilis (14.2%) [22]. Over two-thirds of FSWs in these cities have U.S. clients [23], suggesting the potential for considerable cross-border HIV/STI transmission. High rates of internal migration within Mexico also suggest the potential for rapid transmission of disease throughout the country. As such, innovative program approaches are needed to curtail a bi-national public health crisis.

A growing body of empirical evidence suggests that couple-based research and HIV/STI interventions should be extended to focus on FSWs' intimate, non-commercial relationships. The international, interdisciplinary literature suggests that FSWs are less likely to report condom use with intimate partners than with clients [24-33]. FSWs' intimate partners may engage in high risk behaviors themselves, such as concurrent partnerships with other women and men and injection drug use [34]. Yet few studies have explored the complexity of FSWs' intimate relationships [35-37], including issues relating to drug use [38].

Previously, we showed that a brief behavioral intervention designed to increase FSWs' condom use with male clients reduced STI incidence among FSWs by 40% [39]. Unfortunately, this intervention had no impact on FSWs' condom use with their non-commercial partners, with whom they were twice as likely to have unprotected sex compared with clients [40]. Among 152 FSWs with an intimate partner who were enrolled in an intervention study in Northern Mexico, 50% believed that their partners had concurrent sexual partners, yet 74% reported unprotected vaginal sex with these partners in the past month [41]. In this same sample of women, those with a spouse or common-law partner were twice as likely to have injected drugs in the month prior to the interview [18]. These data suggest that FSWs' *non-commercial *partners may be significant drivers of HIV/STI acquisition. The impact of such behavior is not trivial; when 50% of partnerships in a population are concurrent, the size of the HIV epidemic after 5 years is 10 times larger as under sequential monogamy [42]. Although dozens of studies have been conducted with FSWs in diverse settings, almost none have characterized FSWs' non-commercial partners, who represent a crucial missing link in HIV/STI prevention. This preliminary work highlights the importance of drug and sexual risk behaviors in the context of FSWs' intimate relationships and served as a justification for *Proyecto Parejas*.

### *Proyecto Parejas: *study design and methods

#### Study aims

*Proyecto Parejas *(the "Couples Project" in Spanish) appears to be the first prospective, mixed-methods study of the social context and epidemiology of HIV, STIs, and high risk behaviors among FSWs and their primary, non-commercial male partners in Mexico. The specific aims of the project are to: 1) examine the social context and patterns of high risk sexual and substance using behaviors among FSWs and their non-commercial male partners using a mixed-methods approach; 2) determine prevalence of HIV and specific STIs (syphilis, gonorrhea, and chlamydia) and associated correlates at the individual and partner levels among these couples; 3) prospectively identify predictors of HIV/STI incidence and their attributable risks at the individual and partner level among partners; and 4) use the data from our descriptive study to determine the feasibility of conducting a future behavioral intervention trial among high risk FSWs and their main, non-commercial male partners at the partner or individual level. To meet these aims, the study design requisites recruitment of 100 FSWs and their 100 intimate male partners in both Tijuana and Ciudad Juarez (total n = 400), two Mexico-U.S. border cities with high rates of drug abuse, sex work, and HIV/STIs.

#### Study design

*Proyecto Parejas *is a mixed methods observational study. All couples answer extensive questions in a quantitative survey and provide biological samples for HIV/STI testing. The study design also calls for an initial sub-set of couples at each site to participate in baseline qualitative interviews to provide information about the social context of their relationship. Individual and couple-based qualitative interviews can lend insight into the contextual elements that affect HIV risk behaviors, including the nature of the relationships (e.g., how the partners met, how long they have been together), finances, sex work, sexual practices, drug use, and drug treatment. Interviews are video recorded to assess non-verbal communication and behavior, and audio recorded for transcription and text analysis. Couples receive U.S. $20 total for the joint interview and an additional $20 each for individual interviews.

The quantitative survey covers domains at the individual level, such as socio-demographic and family characteristics, sexual behaviors, substance use, and self-efficacy measures; intrapersonal factors such as depressed mood, self-esteem, and attitudes toward traditional gender roles; and relationship measures, such as relationship stability, perceived risk of sex partners, relationship power, intimate partner violence, communication skills, relationship satisfaction, and condom use norms. All measures are administered using computer-assisted personal interviewing with Questionnaire Development System (QDS) software [43].

After the quantitative survey, nurses draw blood for rapid testing of HIV and syphilis antibodies, and collect urine samples to test for chlamydia and gonorrhea. Confirmatory testing of specimens found positive on rapid HIV and syphilis tests and all testing for chlamydia and gonorrhea are conducted at the San Diego Public Health Laboratory. Positive STI cases receive free treatment based on U.S. and Mexican guidelines and confirmed HIV cases are referred to municipal clinics for free treatment. Individuals are compensated U.S. $20 for the baseline quantitative survey and testing. The study design includes one round of follow-up qualitative interviews, and quantitative follow-up surveys and HIV/STI testing every six months for 24 months.

Our mixed methods prospective design will enable us to meet the study aims in the following ways. To meet Aim 1, we will draw on the semi-structured interview data to examine the social context and patterns of sexual and substance using behaviors within and outside the partnerships. To meet Aim 2, we will use the biologic testing data to determine the prevalence of HIV and specific STIs and associated correlates at the individual and partner level. The primary outcomes for Aim 3 are HIV/STI incident cases over the study period. Over the 24-month follow-up period, we will prospectively examine incidence of HIV, syphilis, gonorrhea, and chlamydia in both partners, and associated predictors and attributable risks at the individual and dyad-level. Importantly, our prospective study design allows us calculate both *relative risks *(i.e., magnitude of risk in the exposed vs. unexposed) at the individual and couple-level as well as *attributable risks *(which in our study is the proportion of incident HIV/STI cases attributable to a specific exposure). Our calculation of attributable risks will help determine the extent to which the outcome (i.e. risk of HIV/STIs) may be attributed to potentially modifiable risk factors of interest, which is critical for informing the most appropriate directions for a future intervention study.

Finally, to meet Aim 4 we will assess the feasibility of conducting a future individual or couple-based intervention with this population. Since there is a paucity of information on the context and behavioral patterns of FSWs and their non-commercial male partners, our observational study is an appropriate prerequisite for a future intervention study. We will draw on both quantitative and qualitative descriptive data to examine potential barriers to interventions such as project attrition, partnership dissolution, and intimate partner violence (IPV). We will evaluate participants' experiences in the project and interest in participating in a subsequent intervention, as well as consider their feedback on key areas that such an intervention could address.

For the purpose of the current paper, we now direct our focus to the methods employed in the successful recruitment of study subjects into *Proyecto Parejas*. This methodological contribution provides a detailed description of the processes involved in the sampling, recruitment, screening, and enrollment of couples into public health research. The remainder of this paper outlines our bi-national efforts to develop and implement a study protocol and offers a practical set of tools that other researchers should find helpful in the study of high risk couples in international and resource-poor settings, with particular relevance to studies of FSWs and their intimate partners.

## Methods/Design

### Study setting and locations

*Tijuana *is the largest Mexico-U.S. border city with an estimated 1.4 million persons [44] and is adjacent to the U.S. city of San Diego, California. Like other large cities in Mexico, Tijuana has a designated *Zona Roja *(red light district) where sex work is tolerated. Sex workers are required to register for a permit in order to work, but in reality, many women continue to exchange sex without such documentation [45]. The most widely cited estimate of the number of FSWs in Tijuana is 9,000 [46].

*Ciudad Juarez*, the largest city in the border state of Chihuahua, has a population of 1.3 million residents [37] and is adjacent to the U.S. city of El Paso, Texas. Like Tijuana, the main industry in Juarez is manufacturing, with a large workforce in *maquiladora *assembly plants. The historical sex work districts in Ciudad Juarez have undergone recent gentrification, and FSWs currently work in several regions of the city where permits are not required. There are an estimated 6,500 FSWs in Ciudad Juarez [47].

### Eligibility criteria

The target population consisted of FSWs and their heterosexual, non-commercial male partners from Tijuana and Ciudad Juarez. Eligibility criteria for women included being at least 18 years old; reporting lifetime use of heroin, cocaine, crack, or methamphetamine; having a stable, non-commercial partner for at least 6 months; reporting any sex with that partner in the 30 days prior to the interview; and having traded sex in the past 30 days. Women were ineligible if they planned to break up with their non-commercial partner, move away, would refuse STI treatment, or if they feared extreme IPV as a result of their participation. Male partners had to be at least 18 years old, be in a non-commercial relationship with an eligible FSW, and report sex with that FSW-partner in the 30 days prior to the screening. Drug use was not an eligibility criterion for men. All screening instruments were programmed in QDS software [43] to automatically exclude participants if they violated these criteria. Participants were not informed why they were ineligible in order to protect the safety of women in cases of extreme IPV and to prevent individuals from informing other potential participants about the study criteria.

### Methodological framework

Couples enrolled in *Proyecto Parejas *were required to pass a two-step screening process: 1) a primary screener, which was first administered to the female partner to check her eligibility as an active sex worker in a steady relationship; and 2) a couple verification screening (CVS), which occurred after eligible women brought their non-commercial male partners into the study offices for a couple-based screening process. During the second step, the CVS was administered to each member of the couple to assess their knowledge of each other and help determine the likelihood that the couple has not falsified information (e.g., in order to obtain compensation). This methodological approach to recruitment and the content of the screening instruments were based on a protocol developed by McMahon and colleagues (2003) [8]. We adapted McMahon's CVS to ensure that it was appropriate for use with FSWs and their intimate partners in Mexico through a collaborative process that drew on input from U.S. study team members trained in epidemiology, psychology, anthropology, biostatics, and health behavior, as well Mexican collaborators trained in medicine, healthcare delivery, psychology, and social work, and field workers who had significant street-based experience working with the local population.

### Gender and power considerations

We were sensitive to the unique ethical considerations that HIV/STI behavioral research with couples poses [8,12], including sensitive topics such as condom use within and outside of the partnership, infidelity, lack of trust, and relationship instability [12]. We also considered gender-based issues of power, control, and dominance in the relationship dynamics [48,49], as ignoring these issues in the recruitment process could result in conflict and potential IPV as an unintended consequence of their participation [12]. For example, interviewers received training to be sensitive to issues of suspected IPV, and we created a safety protocol with input from our multidisciplinary research team, which outlined ways in which staff should respond (e.g., provide referrals for counseling, contact necessary authorities) when participants reported severe IPV. We also carefully designed questions to assess IPV throughout the screening process, as described in the screening sections below.

Like other HIV/STI-related studies with high risk heterosexual couples [8,12], we initiated recruitment through the woman first in order to give her the initial decision-making control to participate in the study and minimize the possibility of coercion by male partners to participate in the study [8]. Contacting the female partners first was also intended to protect their confidentiality as FSWs. During the screening process, women were informed that their partner could find out that they were a sex worker and they were able to decline participation at that time.

### Protocol design and training

Based on the study design conceived by the PI of the project (SS), the U.S. team drafted lists of questions to be included in both screening tools, which were circulated to all team members for feedback and further suggestions. Bilingual team members translated the rough drafts of the instruments into Spanish. All translations were checked by field team members to assure accuracy and appropriateness prior to field testing. Attention was given to incorporating local drug terminology and other street slang (e.g., "What is your/your partner's main *talon *[i.e., local slang for job]?"). The Mexican field staff provided feedback to assess the content of the questions, add additional questions, and identify problematic words or phrases in the translations. All instruments were field tested in Tijuana by three coauthors (JS, AR, AV), who suggested further edits to the questions and overall screening process based on that experience. Field testing was an essential component to the protocol development.

The primary screener and CVS were programmed into multiple laptops at the field offices using QDS software [43]. After programming the initial versions of the instruments using QDS and drafting English and Spanish procedure manuals, the research team held training for field workers from both research sites in Tijuana. Interactive training activities such as role playing allowed fieldworkers to gain experience with the screening instruments and provide each other with feedback on their performance. While many of the field staff at both sites already had extensive experience working with FSWs, training specifically aimed at working with FSWs in relationships provided guidance to address couple-based issues of gender and relationship power. Moreover, conducting an interactive training jointly with fieldworkers from both study sites opened a space to elicit feedback from experienced service providers and research staff prior to launching the project. After the training process, the team further refined the protocol and instruments into the final versions described here.

### Sampling and recruitment procedures

Our sample size was driven by our quantitative and qualitative study aims. To determine the size of the overall sample, statistical power calculations were based on a number of hypotheses associated with the quantitative aims. Estimates for the parameters needed in the power calculations (e.g. sample distributions, percentages, means, standard deviations) were obtained from our previous studies in the region and by consulting the published literature (e.g., [27]). Based on the statistical methods proposed to test our hypotheses, the assumed estimates of 10% annual attrition, 24 months of follow-up, and 0.05 type 1 error, we determined that a baseline sample size of 400 subjects (200 FSWs and 200 non-commercial partners) would be sufficient to achieve at least 80% power in order to detect the smallest effect size of interest. For the qualitative aims, the total number of participants needed for the semi-structured interviews is based on the principle of saturation sampling [50] in that we will continue to recruit participants until it is determined that sufficient saturation of responses to the interview protocol is obtained through an iterative process of data collection and analysis. Saturation, or the ability to predict what informants will say about a particular topic based on that said by previous informants, provides empirical confidence that the sample size is adequate to examine the topics of interest [51].

We employed sampling techniques that are commonly used in studies of hidden populations, including targeted [52] and snowball sampling [53]. Probability sampling techniques are not feasible when working with hidden populations [54]. Because the majority of FSWs working in Tijuana are not registered as sex workers with the municipal health department and Ciudad Juarez does not keep an official registry, the parameters of the FSW populations in both cities are unknown. Respondent driven sampling (RDS) has shown limited effectiveness in recruiting FSW samples [55], and in previous studies of injection drug users in Tijuana and Ciudad Juarez, only 10% of women were successfully recruited using RDS [56,57], despite modifications including providing extra incentives to seeds for recruiting women. Time-location sampling was originally considered for the present study, but was ruled out due to safety concerns in the midst of high levels of drug-related violence, and recruiters were not consistently able to select random venues and times to conduct recruitment. Therefore, targeted [52] and snowball sampling [53] were appropriate in our research context.

Targeted sampling occurred when street-based outreach workers [*promotore/as*] worked in pairs to target areas where sex work and drug use visibly occur (e.g., bars, motels, street corners), and spent time informally observing the women before they unobtrusively approached them to explain the study and assess potential interest in participation. If women were with their partners at the time, female outreach workers explained the study in confidence while the male outreach worker talked to the male about general topics unrelated to the study. Snowball sampling occurred when enrolled FSWs referred other women involved in sex work who they knew from the street or with whom they worked in bars or other establishments. In all cases, outreach workers invited potential female candidates to the research offices to administer the primary screener (see below).

### Step 1: Primary screener

The primary screener assessed FSWs' eligibility for the study and also contained extraneous questions so that women would not be able to guess the eligibility requirements. As indicated in Table 1, examples of extra questions included the number of children they have, if they had children from other partners, and how many times they had crossed the U.S. border in the past year. The screener required about 10 min to complete and women were paid U.S. $5 for their time, regardless of their qualification.

**Table 1:** *Proyecto Parejas *primary screener questions

1.	What is your first name?*
2.	What is your age, in years?
3.	What is your date of birth?
4.	Where do you live?
5.	How long have you lived there?*
6.	How much longer do you think you'll live in (name of the city)?
7.	Have you ever been to the United States?*
8.	When was the last time you were in the United States?*
9.	Do you have any family currently living in the United States?*
10.	If yes, what family members live in the United States? *
11.	Have you ever used an illegal drug?
12.	What drugs have you ever used?
13.	Have you used any drugs in the past three months?*
14.	What drugs have you used in the past three months?*
15.	Have you ever injected an illicit drug?*
16.	Have you ever had an HIV test?*
17.	When was the last time you had an HIV test?*
18.	Have you ever been tested for any sexually transmitted infection such as chlamydia, gonorrhea, or syphilis?*
19.	When was the last time you were tested for any sexually transmitted infection such as chlamydia, gonorrhea, or syphilis?*
20.	If you were to test positive for HIV or a sexually transmitted infection such as chlamydia, gonorrhea, or syphilis, would you agree to receive treatment from a doctor?
21.	Have you ever had sex in exchange for money, drugs, goods or shelter?
22.	When was the first time that you had sex in exchange for money, drugs, goods or shelter?*
23.	When was the last time that you had sex in exchange for money, drugs, goods or shelter?
24.	Are you currently registered with the Municipal Health Services as a Sex worker?*
25.	What is your marital status?*
26.	Do you currently have a spouse or steady partner?
27.	What is your partner's first name?*
28.	How old is your steady partner?*
29.	Is your spouse or steady partner male or female?
30.	How long have you been with your steady partner?
31.	When was the last time you had sex with your spouse or steady partner?
32.	How many children do you and your steady partner have together?*
33.	Who primarily takes care of the children?*
34.	Does your spouse or partner have any children with other women?*
35.	Do you have any plans to end your relationship with your spouse or steady partner in the near future?
36.	If yes, can you please tell me the reason you are thinking about ending your relationship?
37.	Have you ever experienced any physical, sexual, economic, or verbal abuse from your partner? ^++^
	Yes
	No (skip the rest of the section, to Q42)
	Refuse to Answer**
38.	In the past three months, have you experienced physical, sexual, economic, or verbal abuse from your partner? ^++^
	Yes
	No (skip the rest of the section, to Q42)
	Refuse to Answer**
39.	In the past three months, how often have you experienced abuse from your partner? ^++^
	Daily
	Every month
	Occasionally
	Almost never
	Refuse to Answer**
40.	In the past three months, how serious would you describe the level of abuse in your relationship with [your partner]? Can you give me some examples? ^++, +++^
	NONE (for example, has not suffered from any insults, mistreatment or yelling from the partner in the last three months)
	MILD (for example, my partner has insulted, swore, or yelled at me at least once in the past 3 months)
	MODERATE (for example, my partner has pushed, shoved, or slapped me at least once in the past 3 months)
	SEVERE (my partner has punched me, kicked me, or beat me up to the point where I had serious injuries, or has pulled a weapon on me and threatened to kill me one or more times in the past 3 months)
	
	Refuse to Answer**
41.	Are you currently worried that your life is in danger because of abuse from your partner? ^++^
	Yes
	No
	Refuse to Answer**
42.	Are you currently participating in a research study or program with our group?*
43.	Specify other research study or program in which respondent is currently participating*
44.	Would you be willing to come to our office with your spouse or steady partner to see if you are both eligible for a study?

* Questions do not count toward eligibility

** If participant refuses to answer the IPV questions, the interviewer should try to probe for reasons why they refuse to answer, and if they still refuse to answer, they should mark refuse to answer. If the participant refuses to answer any or all of the questions, the interviewer should use their judgment and consult with other field staff to assess the potential risk

^++ ^Questions 37-41 assess intimate partner violence (IPV); IPV questions are also asked of both partners in the CVS

^+++ ^The interviewer should ask for examples of the types of abuse they have experienced and evaluate the level of severity that is most appropriate for their situation from the options listed

### Assessing intimate partner violence (IPV)

Importantly, we took great care to screen for experiences of severe physical or sexual violence or threats to the participant's life within the relationship. After consulting the literature, experts from our team (NE), and the field staff, a multiple-step process was developed to assess the nature and frequency of abuse and exclude those at high risk. A researcher safety protocol provided detailed instructions on how to handle issues of IPV that might arise during the study, and included an extensive list of resources for referrals [9].

Questions about experiences with IPV were embedded in the primary screener to assess the female partner's risk and were repeated during the subsequent couples' screening phase (while the team's violence assessment was designed with female partners in mind, male partners were also screened for IPV in the CVS). As shown in Table 1, IPV was assessed by asking potential female participants whether they had ever experienced any physical, sexual, economic, or verbal abuse from their current partner. If a woman responded affirmatively to this question, she was then asked whether she had experienced any of this abuse in the past three months. Women reporting any abuse in the past three months were then asked the frequency of abuse (e.g., daily, every month, occasionally, and almost never), and if they felt that their life was currently in danger.

An assessment of the severity of IPV was made by asking women how serious they would describe the level of abuse in their relationship and to provide examples. The female partner's self-reported experiences were then compared to a list of examples of different forms of abuse which was constructed from the revised Conflict Tactics Scales [58-60] and suggestions by a staff social worker in Ciudad Juarez who had extensive experience working with survivors of domestic violence. Interviewers used the woman's self-reported information to rate her level of abuse according to three categories: "mild," "moderate," and "severe" (see question 40 in Table 1). Potential participants who reported severe levels of IPV were excluded if the field team determined that study participation would endanger the participant. Potential participants who stated that they were worried that their life was currently in danger because of IPV or who refused to answer that question were automatically excluded from the study. To avoid further endangering the abused partner or reveal study criteria, neither excluded individuals nor their partners were explicitly told why they were excluded from the study. Potential participants who were experiencing severe levels of abuse or worried that their life was in danger were given referrals to local community-based organizations that provide IPV counseling and services.

### Step 2: Couple verification screening (CVS)

Eligible women who passed the primary screener were invited to bring their primary, noncommercial male partners to the study offices for the second step in the screening process, which also served as a means of excluding participants who were less likely to be retained in a prospective study. Step two of the screening process consisted of a 10-15 min CVS that helped confirm each individual's eligibility and verify relationship status by querying individuals about their intimate partner [8]. Interviewers administered questions to elicit personal information about each participant and their partner separately, as described below. The answers provided by each partner in the CVS were then compared by the Field Coordinator to test each partner's knowledge of each other and help confirm the veracity of the relationship. Each partner received U.S. $5 for completing the CVS, regardless of qualification.

The research team and field staff collaborated to adapt and field test a series of questions based on the CVS developed by McMahon et al. (2003) for a U.S.-based population of drug-using couples [8]. With input from all team members, a pool of questions were constructed for field testing in Tijuana in order to assess the acceptability and content of the questions and concordance of responses among known couples. In order to control for situations where one partner was more knowledgeable about the other, the same interviewer gathered data from each partner in succession to better gauge the likelihood that they were a bonafide couple. Interviews were programmed to be administered to one partner immediately after the other, at the end of which a QDS program generated each partner's answers in two printable documents to be compared side by side. This process also allowed field team members to observe the other partner's behavior in the waiting room and informally chat with them, helping to further assess their relationship status and to prevent partners from discussing answers in between interviews.

The *Proyecto Parejas *Couple Verification Screening (CVS) question list shows the final set of questions. We used the majority of questions from the original CVS [8] (indicated in the table with ^d^), but adapted and expanded upon this work. Questions taken directly from the original CVS included "When did you and your partner last have sex?" and "When did you and your partner last do drugs together?" In a similar manner, we added the question "When was the last time you and your partner got into an argument?" We included the original question "How many permanent tattoos, if any, does your partner have on his/her body?" but also created an additional question "What is the biggest tattoo on your/your partner's body?" to try to assess bodily familiarity with each other. We adapted original questions, such as "What is your father's/mother's first name?" to instead assess "How many times per week do you/does your partner talk with your/your partner's immediate family?" Many individuals in this population are migrants from other areas of Mexico and, due their involvement in sex work and drugs, may have limited contact or be estranged from their own families and partners' families. This question assessed the level of contact and involvement with family, even if contact is limited. We also adapted "What is your partner's favorite meal?" to instead assess "The last meal you shared together with your partner, what did you eat?" and asked additional sub-questions: "When was that meal?" "What was it?" and "Where did you eat?" These questions were changed because severely drug dependent participants have indicated to us that food is sometimes a relatively low priority and limited income may be reserved primarily for drugs, suggesting that favorite foods are a luxury commodity infrequently consumed or discussed. As such, the series of questions about food in our CVS was modified to be more proximate in nature.

#### Proyecto Parejas Couple Verification Screening (CVS) question list^c^

QUESTIONS ASKED OF ALL INDIVIDUALS:

■ Initial questions re-assess eligibility criteria and intimate partner violence (from the primary screener)

■ What is **your/your partner's **date of birth? ^d^

■ How old **are you/is your partner**?

■ Please tell me which drugs **you/your partner **are currently using.

■ Where did you and your partner meet each other for the first time? (Probe for a specific location, not just a city or "on the street")

■ What year and month was it when you met your partner?

POOL OF QUESTIONS FROM SIX DIFFERENT CVS VERSIONS:

■ What is **your/your partner's **steady job?

■ About how many hours do **you/does your partner **work each day?

■ At what time do **you/your partner **usually start work?

■ At what time do **you/your partner **usually finish work?

■ Where (physical location) do **you/your partner **go most often to use drugs?

■ How old did you tell your partner you are? How old did your partner tell you she/he is?

■ How many permanent tattoos do you have on your body? How many permanent tattoos does your partner have on his/her body? ^d^

■ How many children live with **you/your partner **right now?

■ On what part of the body do **you/your partner **have your largest scar?

■ How many daughters do **you/does your partner **have?

■ How many sons do **you/does your partner **have?

■ To which *connecta *or *picadero*^a^ do **you/your partner **usually go?

■ Where were **you/your partner **born (city, state and country)? ^d^

■ What is the biggest tattoo on **your/your partner's **body?

■ When you and your partner met for the first time, who made the first move?

■ What is the name of one of **your/your partner's **friends (someone with whom you spend the most time)?

■ What does your partner call you (a nickname)? What do you call your partner (a nickname)?

■ Where do **you/your partner **live most of the time?

■ What do **you/your partner **have tattooed on your back?

■ The last meal you shared together with your partner, what did you eat?

When was that meal? What was it? Where did you eat?

■ How many times per week do **you/your partner **talk with your/your partner's immediate family?

■ When was the last time you and your partner got into an argument?

■ How old are you? How old is your partner? ^d^

■ What is **your/your partner's **main *talon*^*b*^*?*

■ In what area or location **do you/does your partner **spend most of the time when trying to earn money? (probe for a specific bar, motel corner, street)

■ The last time that you and your partner slept in the same bed together, who slept closer to the door? ^d^

■ How many brothers **do you/does your partner **have? ^d^

■ How many sisters **do you/does your partner **have? ^d^

■ If your partner needed to call you, what number would he/she call first? If you needed to call your partner, what number would you call first?

■ Where do **your parents/your partner's parents **live?

■ If you had the opportunity to travel, where would you like to go (probe for a specific location)? If your partner had the opportunity to travel, where would he/she like to go?

■ Do **you/does your partner **inject drugs?

■ In what part of the body do **you/your partner **usually inject?

■ On what date did you last have sex with your partner? ^d ^On that day, at what time did you have sex with your partner?

■ Where did **you/your partner **sleep last night?

■ Where did **you/your partner **sleep the night before last?

■ In case of an emergency or illness, where would **you/your partner **go?

■ When was the last time **you/your partner **were picked up by the police and put in jail?

■ Where do **you/your partner **sleep most of the time (probe for specific location)?

■ If your partner wasn't at home and you needed to find him/her, what's the first place you would go look for him/her? If you weren't at home and your partner needed to find you, what's the first place he/she would go look for you? (Probe for specific name, especially if response is a bar or on the street)

■ Who helps **you/your partner **when you are sick?

■ Do you have someone you can call to get you out of jail/when you're picked up by the police? Who? Does your partner have someone he/she can call to get him/her of jail/when he/she is picked up by the police? Who?

FINAL QUESTIONS FOR INTERVIEWER:

■ On a scale of 1 to 10, with one being not at all confident to 10 being perfectly confident, how confident are you that this is an actual couple?

■ Interviewer notes/comments:

We also created new questions that drew from the team's familiarity and experience working with the population in the local cultural and socioeconomic context of the Mexico-U.S. border region. For example, because nicknames are very common in the study population, frequently used in the local street culture as well as terms of endearment in close relationships, we asked: "What does your partner call you (a nickname)? What do you call your partner (a nickname)?" The team also designed questions relevant to the daily hardships faced by the population. For example, because much of the population spends a significant amount of time on the streets and in public places to informally earn money, we assessed "In what area or location do you/does your partner spend most of the time when you are trying to earn money? (probe for a specific bar, motel corner, street)" and "If your partner wasn't at home and you needed to find him/her, what's the first place you would go look for him/her? If you weren't at home and your partner needed to find you, what's the first place he/she would go look for you? (Probe for specific name, especially if response is a bar or on the street)." Other questions acknowledged drug involvement, such as "To which *connecta *[place to purchase drugs] or *picadero *[shooting gallery] do you/your partner usually go?" These questions also provided the field teams with current information on the constantly changing local drug markets. Finally, because this population faces frequent harassment by the police, the question "When was the last time you/your partner were picked up by the police and put in jail?" was appropriate in the local context.

Ultimately, in order to render the eligibility criteria less evident and reduce the likelihood that individuals could rehearse their answers to a known set of questions, six versions of the CVS were programmed into QDS, which randomly generated one of the versions for each couple who underwent screening. Each CVS version contained eligibility questions, core relationship questions that were asked of all couples (e.g., "Where did you and your partner meet each other for the first time?"), and a random series of questions drawn from the list in *Proyecto Parejas *Couple Verification Screening (CVS) question list.

Like McMahon's protocol (2003) [8], we decided against having a hard rule that partners' answers had to match exactly. Instead, we took proximity of responses into account (e.g., partners providing birthdates for each other that were technically incorrect but matched closely). Whenever possible, the field team also drew on their observations and personal knowledge of the couples to make a decision about their eligibility.

Enrollment was an ongoing process that involved field staff at both sites in Mexico and checks by statisticians in San Diego. Decisions to enroll couples were made in the field on a case-by-case basis that considered the totality of evidence from the CVS responses and staff observations and knowledge. Interviewers documented the reasons why couples were disqualified and, if excluded, individuals were not informed why they were excluded or whose responses led to exclusion. Enrollment and disqualification were reviewed biweekly by Field Coordinators in both sites and statisticians in San Diego in a process of routine data collection and quality control. If eligible, staff reviewed study procedures and potential risks so that each partner could provide written informed consent. All study protocols were approved by the University of California, San Diego's Human Subjects Research Protections Program and the institutional review boards of the Hospital General and El Colegio de la Frontera Norte in Tijuana and the Universidad Autónoma de Ciudad Juárez.

## Results

### Screening and enrollment results

In total, 335 women were screened, of whom 245 (73.1%) were eligible. Table 2 lists the reasons that women were disqualified based on the primary screener (n = 90 disqualified, 26.9% of those who completed screeners). The most common reason for ineligibility was failure to meet the criteria for "hard" drug use (10.4%), which we defined a priori as any lifetime use of heroin, methamphetamine, or cocaine or crack. No recent sex work (6.9%), having imminent plans to break up with the steady partner (6.0%), and reporting no recent sex with the steady partner (5.1%) were the next most common disqualifiers. The IPV questions excluded 14 women (4.2%) at the primary screener phase. Staff reported no incidents requiring enactment of the safety protocol.

**Table 2:** Reasons for disqualification from *Proyecto Parejas *in the primary screener,* n = 90

Disqualification Reasons	Total (n, %)
No lifetime use of cocaine, methamphetamine, or heroin	35 (10.4%)
No sex work in last month	23 (6.9%)
Plans to break up with partner	20 (6.0%)
No sex with partner in last month	17 (5.1%)
Worried about intimate partner violence (IPV)	14 (4.2%)
Never exchanged sex for money, drugs, or other items	11 (3.3%)
Unwilling to bring in partner for screening	10 (3.0%)
Relationship < 6 months	9 (2.7%)
Plans to move in next 18 months	3 (0.9%)
Would refuse treatment for STIs	2 (0.6%)
Does not have a steady partner	2 (0.6%)
Primary partner is female	2 (0.6%)
Age < 18	1 (0.3%)

* Numbers do not add to 100% because women could be excluded for more than one reason

**Figure 1:**
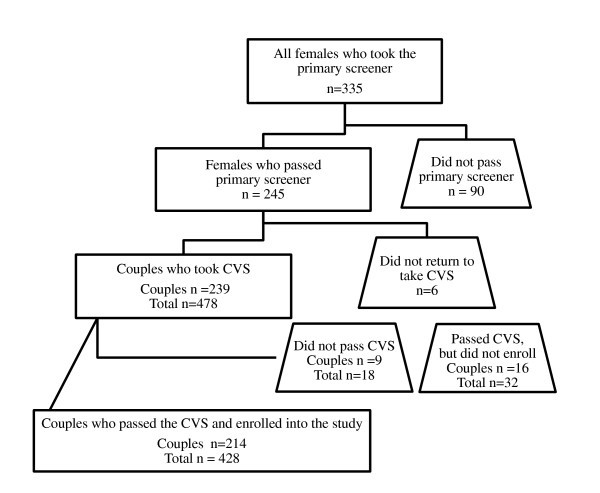
**Flowchart of Proyecto Parejas recruitment in Tijuana and Ciudad Juarez, Mexico**.

Table 3 depicts all couples who were screened with the CVS: of the 239 total couples, 230 (96.2%) passed and were eligible to enroll in the study. Of the nine couples (3.8%) who did not pass the CVS stage, two couples were excluded because of the male partner's concern over IPV and seven were determined not to be real couples based on the excessive discordance of partners' responses in the CVS and the staff's observations of the potential participants. Figure 1 depicts a flowchart of the overall screening processes in Tijuana and Ciudad Juarez. In addition to the couples excluded by the CVS, another 16 couples passed the CVS but did not enroll in the study. Of those couples, 14 did not return for a baseline interview and two male partners in Ciudad Juarez died after participating in baseline qualitative interviews but before the baseline quantitative survey and HIV/STI testing were administered to complete their enrollment; one died from a drug overdose and the other was a homicide victim. In total, 214 couples were successfully enrolled in *Proyecto Parejas*.

**Table 3:** *Proyecto Parejas *Couple Verification Screening (CVS), n = 239 couples

CVS Eligibility Status	Total (n, %)
Qualified for the study	230 (96.2%)
Excluded from the study	9 (3.8%)
Worried about IPV	2 (0.8%)
Determined not a real couple	7 (2.9%)

## Discussion

Our study supports the approach to recruit, screen, and enroll high risk couples outlined by McMahon and colleagues (2003) [8] and extends these protocols to the study of FSWs and their intimate, non-commercial partners in a resource poor setting. Based on this experience, we offer several suggestions with applicability to other studies of high risk couples in diverse social and cultural contexts. Main points of reflection center on collaborative decision making and considering the local context in protocol development; strategies to mitigate participant risk, particularly regarding possible IPV; and suggestions for assessing the methodological rigor of the screening process of couple-based studies.

Our adaption of McMahon et al's protocol [8] is innovative in several ways. First, while self-reported relationship status is typically used in couple-based studies and may be sufficient in many settings, prior experience working with drug-involved FSWs who live and work along the Mexico-U.S. border suggested that they were street savvy and that verifying partner status would provide additional oversight to the screening process. Indeed, in Tijuana, a member of a "couple" excluded by the CVS angrily complained to the Field Coordinator that the questions differed from what another enrolled FSWs told them to expect. We generated a large enough pool of questions to create multiple versions of the CVS and thwart potential participants posing as a couple. Moreover, our questions were developed with multiple perspectives from team members to assure cultural relevance and sensitivity to the social context of this border population, including issues related to family, informal economies, violence, local drug markets, and law enforcement activity.

Collaborative efforts are vital in protocol construction when working in resource poor and international settings. Drawing on formative work in the region, eliciting input from the entire research team and field staff, and field testing the individual measures and flow of the entire process proved vital to the final protocol development. Eliciting a wide range of input ensured the inclusion of multiple viewpoints and different academic perspectives, thus strengthening the final protocol. Fieldworkers, particularly those with extensive experience in other research projects with similar populations, know the local cultural and socioeconomic contexts and can make valuable contributions to protocol and instrument design, including assessing the appropriateness of questions. Local input is imperative in adapting measures for relevance within the linguistic, socio-cultural, and economic contexts of unique geographic regions and study populations [61-63]. The protocols and instruments for our study benefitted greatly from this collaborative process.

Field testing the screening instruments and overall enrollment process was also critical to the successful implementation of our study. Based on experience field testing the CVS with known couples, we modified the original protocol and decided to use the same interviewer to administer the questions to each partner sequentially to try to get a better sense of whether or not the couple was legitimate. We suggest that in certain contexts a single interviewer, regardless of gender matching with the interviewee, who can probe for consistency of responses may improve the more quantitative approach developed by McMahon et al. (2003) [8]. If a single interviewer is well trained in a structured approach and gains some experience with the population, project purpose, and screening questions, then the validity and reliability of the screening instruments may be improved.

In addition, the CVS screening questions required both closed and open-ended answers. The majority of the questions were closed-ended with a list of options based on formative fieldwork. Eliciting open-ended answers, however, meant that interviewers administering the CVS could probe each partner for specific details to try to better assess the veracity of the information provided. Open-ended questions can also generate emergent data for other analyses, such as assessing the locations where participants purchase drugs in a constantly changing local drug market. Open and close-ended questions are equally easy to assess for concordance in the field. Close-ended questions render ad hoc analyses much easier, but including a few open-ended questions can personalize the interview and add another layer of reflection to the screening process that later can be quantified for analyses.

Within a nexus of sex work, drug use, and HIV/STI risk in places like the Mexico-U.S. border region, everyday violence can become normalized and internalized [64,65]. As such, research protocols must be sensitive to screen for cases of potential violence. Among FSWs, it is important to develop specific screening questions that clearly define cases of IPV which should be excluded because participation in a study might place them at further risk. Based on the Revised Conflict Tactics Scales [58-60] and input from the field teams, our screening tools clearly assessed the timeframe, frequency, type, and severity of violence experienced by participants within their intimate relationships, and generated automatic disqualification from the study based on key responses. Staff were also trained to screen out other cases of IPV on a case-by-case basis if need be, but this situation did not occur at either site.

We also followed McMahon et al's (2003) [8] suggestion to recruit through women first in order to provide her with greater decision making power, screen out cases of extreme IPV, and reduce female participants' risk. This approach, however, may bias the sample in favor of "less risky" couples. A recent study found that among FSWs enrolled in an HIV intervention who reported having steady partners, those who reported IPV were significantly more likely to report that their partners engaged in known HIV risk factors such as injection drug use and having had sex with another partner while in their current relationship than were FSWs who did not report IPV [66]. It is worth clarifying, however, that we did not screen out couples reporting any IPV, but only those reporting extreme violence that could be life threatening. Overall, 4.2% of women who took the primary screener and 0.8% of couples who were administered the CVS were excluded due to extreme IPV. Thus, while our sample may underestimate risk in this population, the relatively small numbers of participants excluded based on this criteria suggest that the impact on our findings will be minimal.

Regardless, researchers have a responsibility to carefully assess the prevalence of IPV in the population to make an informed decision that protects the safety of the participants. Staff and participant safety protocols for emergent cases of violence and a list of local referrals for psychological counseling and other forms of assistance should also be developed. While our protocols were originally designed with the female participant's safety in mind, it is worth noting that during the CVS stage, two couples (one at each site) were screened out due to *male *partner concerns about IPV, but no additional couples were screened out due to female concerns about IPV. These results suggest the utility of individually screening the female partner for IPV prior to male partner involvement in the process, and serve as a reminder that males are not immune from experiencing partner violence within the context of an intimate relationship.

As advocated by Witte and colleagues (2004) [12], recruitment protocols should be carefully defined and codified in a manual of procedures. Although we created such a manual and conducted extensive training to standardize procedures across sites, we also recommend that the research team view recruitment and screening as a process of multiple components, which allows for a certain level of flexibility in the field. It was important to strictly adhere to the study inclusion and exclusion criteria, which were checked at each phase of screening. It was equally important, however, to enroll couples based on the totality of evidence, including the concordance of couples' answers about each other in the CVS and the local field staff's knowledge and observations of the couples whenever possible. Instead of opting for hard cutoffs in matching partners' answers with each other on the CVS, this process took into account proximity of answers. Overall, nine couples (3.8%) were disqualified based on the CVS. In seven instances, couples were not automatically disqualified by the computer programming of the CVS, but rather by field staff who determined that their answers were too discordant and their interpersonal interactions too awkward or distant to indicate that they were a real couple. The astute judgments of well trained and culturally attuned staff who have insight into the local social context are an invaluable part of the recruitment process.

Finally, while this protocol appeared to have excluded those who did not qualify for the study, more sophisticated analyses are needed to determine the effectiveness of these screening tools [12]. Researchers could test the effectiveness of individual questions in the CVS by determining which ones show higher concordance of responses by partners. Another option would be to calculate similarity coefficients to assess how "close" couples measure in their responses [67]. Coefficients could be used to test for differences between couples who were included versus excluded from the study, and assess differences between couples whose score signaled high similarity versus those whose scores were not as close. Such analyses would lend methodological rigor to the recruitment processes of couple-based studies and help other researchers adapt their own protocols to include measures grounded in empirical evidence. Comparisons of couples' CVS scores to their responses to other quantitative and qualitative instruments used in the study could also help contribute evidence regarding the validity of screening instruments and procedures. Couples should also be observed prospectively to assess correlations between initial CVS scores, relationship stability, and dissolution.

## Conclusion

Attention to the complex social dynamics of intimate relationships and their influence on sexual and drug-related risk behaviors promises to advance public health interventions beyond the individualistic approaches that have thus far failed to contain the HIV epidemic. This study illustrates the feasibility of conducting public health research with high risk couples in a resource poor setting. With multiple sources of input from researchers and field staff, attentiveness to the local context, field testing, and a design that incorporates multiple sources of data to verify couple eligibility, feasible and appropriate study protocols can successfully enroll FSWs and their intimate, non-commercial partners into HIV/STI prevention studies. Other researchers are encouraged to adapt, refine, and improve such couple-based protocols as part of a continually expanding interest in relationship-based public health research and interventions.

## Endnotes

^a^A *connecta *is local slang for a place to purchase drugs and a *picadero *is a shooting gallery

^b^A *talon *is local slang for a job, often jobs that are part of an informal economy such as washing cars that are waiting in line at traffic lights or at the border

^c^Questions worded with "you/your partner" indicate the same question was asked first about the participant and then about their partner

^d^Questions were taken from McMahon et al. (2003)

## Competing interests

The authors declare that they have no competing interests.

## Authors' contributions

JLS wrote the initial draft of the manuscript and helped design and field test the measures and process; AMR and AV helped design and field test the measures and contributed content and revised the manuscript; DA contributed content, revised the manuscript, and ran the analyses; NE designed the violence protocols and contributed content and revised the manuscript; MGR, GM, TLP, and MDU contributed content and revised the manuscript; and SAS conceived of the study design, and contributed content, revised, and provided final approval of the manuscript. All authors read and approved the final manuscript.

## Pre-publication history

The pre-publication history for this paper can be accessed here:

http://www.biomedcentral.com/1471-2458/12/136/prepub

## References

[B1] Coates TJ Richter L Caceres C Behavioural strategies to reduce HIV transmission: how to make them work better Lancet 2008 372 9639 669 684 10.1016/S0140-6736(08)60886-7 18687459 PMC2702246

[B2] Office of AIDS Research FY 2010 Trans-NIH Plan for HIV-Related Research 2010 Washington: Department of Health and Human Services

[B3] El-Bassel N Jemmott JB Landis JR Pequegnat W Wingood GM Wyatt GE Bellamy SL National institute of mental health multisite Eban HIV/std prevention intervention for african american HIV serodiscordant couples: a cluster randomized trial Arch Intern Med 2010 170 17 1594 1601 10.1001/archinternmed.2010.261 20625011 PMC4011550

[B4] El-Bassel N Witte SS Gilbert L Wu E Chang M Hill J Steinglass P The efficacy of a relationship-based HIV/STD prevention program for heterosexual couples Am J Public Health 2003 93 6 963 10.2105/AJPH.93.6.963 12773363 PMC1447878

[B5] Powers MB Vedel E Emmelkamp PMG Behavioral couples therapy (BCT) for alcohol and drug use disorders: a meta-analysis Clin Psychol Rev 2008 28 6 952 962 10.1016/j.cpr.2008.02.002 18374464

[B6] Winters J Fals-Stewart W O'Farrell TJ Birchler GR Kelley ML Behavioral couples therapy for female substance-abusing patients: Effects on substance use and relationship adjustment J Consult Clin Psychol 2002 70 2 344 11952192 10.1037//0022-006x.70.2.344

[B7] Burton J Darbes LA Operario D Couples-focused behavioral interventions for prevention of HIV: systematic review of the state of evidence AIDS Behav 2010 14 1 1 10 10.1007/s10461-008-9471-4 18843530 PMC3640442

[B8] McMahon JM Tortu S Torres L Pouget ER Hamid R Recruitment of heterosexual couples in public health research: a study protocol BMC Medical Res Methodology 2003 3 1 24 10.1186/1471-2288-3-24 PMC272932 14594457

[B9] El-Bassel N Witte SS Gilbert L Sormanti M Moreno C Pereira L Elam E Steinglass P HIV prevention for intimate couples: a relationship-based model Families, Systems Health 2002 19 4 379 395

[B10] El-Bassel N Witte SS Gilbert L Wu E Chang M Hill J Steinglass P Long-term effects of an HIV/STI sexual risk reduction intervention for heterosexual couples AIDS Behav 2005 9 1 1 13 10.1007/s10461-005-1677-0 15812609

[B11] NIMH Multisite HIV/STD Prevention Trial for African American Couples Group STD Prevention Trial for African American Couples Group. Methodological overview of an African American couple-based HIV/STD prevention trial J Acquir Immune Defic Syndr 2008 49 suppl 1 S3 S14 18724188 10.1097/QAI.0b013e3181842570 PMC2910525

[B12] Witte SS El-Bassel N Gilbert L Wu E Chang M Steinglass P Recruitment of minority women and their main sexual partners in an HIV/STI prevention trial J Women's Health 2004 13 10 1137 1147 10.1089/jwh.2004.13.1137 15650347

[B13] Wu E El-Bassel N Witte SS Gilbert L Chang M Morse P Enrollment of minority women and their main sexual partners in an HIV/STI prevention trial AIDS Educ Prev 2005 17 1 41 52 10.1521/aeap.17.1.41.58685 15843109

[B14] Pappas-DeLuca KA Kraft JM Edwards SL Casillas A Harvey SM Huszti HC Recruiting and retaining couples for an HIV prevention intervention: lessons learned from the PARTNERS project Heal Educ Res 2006 21 5 611 10.1093/her/cyl030 16766606

[B15] Preloran HM Browner CH Lieber E Strategies for motivating Latino couples' participation in qualitative health research and their effects on sample construction Am J Public Health 2001 91 11 1832 10.2105/AJPH.91.11.1832 11684612 PMC1446887

[B16] El-Bassel N Gilbert L Wu E Witte SS Chang M Hill J Remien RH Couple-based HIV prevention for low-income drug users from New York City: a randomized controlled trial to reduce dual risk JAIDS 2011 58 2 198 206 10.1097/QAI.0b013e318229eab1 21725249 PMC5870871

[B17] Singer M Huertas E Scott G Am I my brother's keeper? A case study of the responsibilities of research Hum Organ 2000 59 4 389 400

[B18] Rekart ML Sex-work harm reduction Lancet 2006 366 9503 2123 2134 10.1016/S0140-6736(05)67732-X 16360791

[B19] Cusick L Widening the harm reduction agenda: from drug use to sex work International J Drug Policy 2006 17 1 3 11 10.1016/j.drugpo.2005.12.002

[B20] Ghys PD Jenkins C Pisani E HIV surveillance among female sex workers AIDS 2001 15 Suppl 3 S33 S40 11421180 10.1097/00002030-200104003-00005

[B21] Teela S A continuum of risk? The management of health, physical and emotional risks by female sex workers Sociology Health Illness 2004 26 5 557 574 10.1111/j.0141-9889.2004.00405.x 15283777

[B22] Patterson TL Semple SJ Staines H Lozada R Orozovich P Bucardo J Philbin MM Pu M Fraga M Amaro H Prevalence and correlates of HIV infection among female sex workers in 2 Mexico-US border cities J Infect Dis 2008 197 5 728 732 10.1086/527379 18260766 PMC2872174

[B23] Strathdee SA Lozada R Semple SJ Crozovich P Pu M Staines-Orozco H Fraga-Vallejo M Amaro H Delatorre A Magis-Rodriguez C Characteristics of female sex workers with US clients in two Mexico-US border cities Sex Transm Dis 2008 35 3 263 268 10.1097/OLQ.0b013e31815b0 18032996 PMC2737364

[B24] Green ST Goldberg DJ Female streetworker-prostitutes in Glasgow: a descriptive study of their lifestyle AIDS Care 1993 5 3 321 10.1080/09540129308258615 8218467

[B25] Hong Y Li X Behavioral studies of female sex workers in China: a literature review and recommendation for future research AIDS Behav 2008 12 4 623 636 10.1007/s10461-007-9287-7 17694431

[B26] Jackson LA Augusta-Scott T Burwash-Brennan M Karabanow J Robertson K Sowinski B Intimate relationships and women involved in the sex trade: perceptions and experiences of inclusion and exclusion Health 2009 13 1 25 46 10.1177/1363459308097359 19103714

[B27] Jamner S Wolitski RJ Corby NH Fishbein M Using the theory of planned behavior to predict intention to use condoms among female sex workers Psychol Heal 1998 13 2 187 205 10.1080/08870449808406746

[B28] Kerrigan D Ellen JM Moreno L Rosario S Katz J Celentano DD Sweat M Environmental-structural factors significantly associated with consistent condom use among female sex workers in the Dominican Republic AIDS 2003 17 3 415 423 10.1097/00002030-200302140-00016 12556696

[B29] Mills S Benjarattanaporn P Bennett A RACHITTA NAP Sundhagul D Trongsawad P Gregorich S Hearst N Mandel J HIV risk behavioral surveillance in Bangkok, Thailand: sexual behavior trends among eight population groups AIDS 1997 11 Suppl 1 S43 S51 9376100

[B30] Murray L Moreno L Rosario S Ellen J Sweat M Kerrigan D The role of relationship intimacy in consistent condom use among female sex workers and their regular paying partners in the Dominican Republic AIDS Behav 2007 11 3 463 470 10.1007/s10461-006-9184-5 17096198

[B31] Philpot C Harcourt C Edwards J A survey of female prostitutes at risk of HIV infection and other sexually transmissible diseases Genitourinary Med 1991 67 5 384 388 10.1136/sti.67.5.384 PMC1194737 1743710

[B32] Rosenthal D Oanh TTK Listening to female sex workers in Vietnam: influences on safe-sex practices with clients and partners Sex Health 2006 3 1 21 32 10.1071/SH05040 16607971

[B33] Sanders T The condom as psychological barrier: female sex workers and emotional management Feminism Psychology 2002 12 4 561 566 10.1177/0959353502012004016

[B34] Mastro TD De Vincenzi I Probabilities of sexual HIV-1 transmission AIDS 1996 10 Suppl A S75 S82 8883613 10.1097/00002030-199601001-00011

[B35] Warr DJ Pyett PM Difficult relations: sex work, love and intimacy Sociology Health Illness 1999 21 3 290 309 10.1111/1467-9566.00157

[B36] Jackson LA Bennett CG Sowinski BA Stress in the sex trade and beyond: women working in the sex trade talk about the emotional stressors in their working and home lives Critical Public Health 2007 17 3 257 271 10.1080/09581590701549535

[B37] Stoebenau K Hindin MJ Nathanson CA Rakotoarison PG Razafintsalama V "... But then he became my Sipa": the implications of relationship fluidity for condom use among women sex workers in Antananarivo, Madagascar Am J Public Health 2009 99 5 811 819 10.2105/AJPH.2007.118422 19299685 PMC2667855

[B38] Shannon K Kerr T Allinott S Chettiar J Shoveller J Tyndall MW Social and structural violence and power relations in mitigating HIV risk of drug-using women in survival sex work Social Sci Med 2008 66 4 911 921 10.1016/j.socscimed.2007.11.008 18155336

[B39] Patterson TL Mausbach B Lozada R Staines-Orozco H Semple SJ Fraga-Vallejo M Orozovich P Abramovitz D de la Torre A Amaro H Efficacy of a brief behavioral intervention to promote condom use among female sex workers in Tijuana and Ciudad Juarez, Mexico Am J Public Health 2008 98 11 2051 10.2105/AJPH.2007.130096 18799768 PMC2633868

[B40] Ulibarri MD Strathdee SA Lozada R Staines-Orozco HS Abramovitz D Semple SJ Martinez G Patterson TL Condom use among female sex workers and their noncommercial partners: effects of a sexual risk intervention in two Mexican cities Int J STD AIDS in press 10.1258/ijsa.2011.011184 PMC3353829 22581944

[B41] Ojeda VD Strathdee SA Lozada R Rusch MLA Fraga M Orozovich P Magis-Rodriguez C De La Torre A Amaro H Cornelius W Associations between migrant status and sexually transmitted infections among female sex workers in Tijuana, Mexico Sex Transm Infect 2009 85 6 420 426 10.1136/sti.2008.032979 19188211 PMC2819150

[B42] Morris M Kretzschmar M Concurrent partnerships and the spread of HIV AIDS 1997 11 5 641 648 10.1097/00002030-199705000-00012 9108946

[B43] Questionnaire Development System 2010 Bethesda: NOVA Research Company

[B44] INEGI XII Censo General de Poblacion y Vivienda 2005 INEGI

[B45] Sirotin N Strathdee SA Lozada R Nguyen L Gallardo M Vera A Patterson TL A comparison of registered and unregistered female sex workers in Tijuana, Mexico Public Health Rep 2010 125 Suppl 4 101 109 20626197 10.1177/00333549101250S414 PMC2882980

[B46] Brouwer KC Strathdee SA Magis-Rodríguez C Bravo-García E Gayet C Patterson TL Bertozzi SM Hogg RS Estimated numbers of men and women infected with HIV/AIDS in Tijuana, Mexico J Urban Health 2006 83 2 299 307 10.1007/s11524-005-9027-0 16736378 PMC2527171

[B47] Valdez A Cepeda A Kaplan CD Codina E Sex work, high-risk sexual behavior and injecting drug use on the US-México border: Ciudad Juárez, Chihuahua 2002 Houston: Office for Drug and Social Policy Research, University of Houston

[B48] Amaro H Love, sex and power: considering women's realities in HIV prevention Am Psychol 1995 50 6 437 447 7598292 10.1037//0003-066x.50.6.437

[B49] Pulerwitz J Amaro H De Jong W Gortmaker SL Rudd R Relationship power, condom use and HIV risk among women in the USA AIDS Care 2002 14 6 789 800 10.1080/0954012021000031868 12511212

[B50] Guba E Lincoln Y Fourth Generation Evaluation 1989 Newbury Park: Sage

[B51] Guest G Bunce A Johnson L How many interviews are enough? Field Methods 2006 18 1 59 82 10.1177/1525822X05279903

[B52] Watters J Biernacki P Targeted sampling: options for the study of hidden populations Soc Probl 1989 36 4 416 430 10.1525/sp.1989.36.4.03a00070

[B53] Biernacki P Waldorf D Snowball sampling: problems and techniques of chain referral sampling Sociological Methods Res 1981 10 2 141 163

[B54] Magnani R Sabin K Saidel T Heckathorn D Review of sampling hard-to-reach and hidden populations for HIV surveillance AIDS 2005 19 Suppl 2 S67 72 15930843 10.1097/01.aids.0000172879.20628.e1

[B55] Simic M Johnston LG Platt L Baros S Andjelkovic V Novotny T Rhodes T Exploring barriers to 'respondent driven sampling' in sex worker and drug-injecting sex worker populations in Eastern Europe J Urban Health 2006 83 Suppl 1 6 15 10.1007/s11524-006-9098-6 PMC1705510 17109206

[B56] Frost SDW Brouwer KC Firestone Cruz MA Ramos R Ramos ME Lozada RM Magis-Rodriguez C Strathdee SA Respondent-driven sampling of injection drug users in two US-Mexico border cities: recruitment dynamics and impact on estimates of HIV and syphilis prevalence J Urban Health 2006 83 Suppl 1 83 97 10.1007/s11524-006-9104-z PMC1705507 17072761

[B57] Strathdee SA Lozada R Pollini RA Brouwer KC Mantsios A Abramovitz DA Rhodes T Latkin CA Loza O Alvelais J Individual, social, and environmental influences associated with HIV infection among injection drug users in Tijuana, Mexico J Acquir Immune Defic Syndr 2008 47 3 369 376 10.1097/QAI.0b013e318160d5ae 18176320 PMC2752692

[B58] Straus MA Cross-cultural reliability and validity of the revised conflict tactics scales: a study of university student dating couples in 17 nations Cross-Cultural Res 2004 38 4 407 432 10.1177/1069397104269543

[B59] Straus MA Douglas EM A short form of the Revised Conflict Tactics Scales, and typologies for severity and mutuality Violence and Victims 2004 19 5 507 520 10.1891/vivi.19.5.507.63686 15844722

[B60] Straus MA Hamby SL Boney-McCoy S Sugarman DB The revised conflict tactics scales (CTS2): Development and preliminary psychometric data J Fam Issues 1996 17 3 283 10.1177/019251396017003001

[B61] Collins D Pretesting survey instruments: an overview of cognitive methods Qual Life Res 2003 12 3 229 238 10.1023/A:1023254226592 12769135

[B62] Pasick RJ Stewart SL Bird JA D'Onofrio CN Quality of data in multiethnic health surveys Public Health Rep 2001 116 Suppl 1 223 243 11889288 10.1093/phr/116.S1.223 PMC1913670

[B63] Warnecke RB Johnson TP Chávez N Sudman S O'Rourke DP Lacey L Horm J Improving question wording in surveys of culturally diverse populations* 1 Ann Epidemiol 1997 7 5 334 342 10.1016/S1047-2797(97)00030-6 9250628

[B64] Bourgois P Prince B Moss A The everyday violence of hepatitis C among young women who inject drugs in San Francisco Hum Organ 2004 63 3 253 264 16685288 10.17730/humo.63.3.h1phxbhrb7m4mlv0 PMC1458969

[B65] Scheper-Hughes N Death without weeping: The violence of everyday life in Brazil 1993 Berkeley: University of California Press

[B66] Ulibarri MD Strathdee SA Lozada R Magis-Rodriguez C Amaro H O'Campo P Patterson T Intimate partner violence among female sex workers in Two Mexico-U.S. border cities: partner characteristics and HIV risk behaviors as correlates of abuse Psychol Trauma: Theory Res Pract Policy 2010 2 4 318 325 10.1037/a0017500 PMC3083072 21532933

[B67] Gower JC A general coefficient of similarity and some of its properties Biometrics 1971 27 4 857 871 10.2307/2528823

